# Anti-inflammatory activity of α-tomatine *via* inhibition of the MAPK and NF-κB signaling pathway *in vitro* and *ex vivo*

**DOI:** 10.7150/ijms.118250

**Published:** 2026-01-01

**Authors:** Chien-Wei Chen, Chu-Chun Hsieh, Yi-Ting Hsieh, Ming-Ju Lin, Po-Hao Chiu, Chi-Chang Juan, Fu-Kong Lieu, Shyi-Wu Wang, Paulus S. Wang

**Affiliations:** 1 Department of Physical Education, Health, and Recreation, Teachers College, National Chiayi University, Chiayi, Taiwan, ROC.; 2 Institutes of Physiology, College of Medicine, National Yang Ming Chiao Tung University, Taipei, Taiwan, ROC.; 3 Department of Educational Management, National Taipei University of Education, Taipei, Taiwan, ROC.; 4 Department of Medical Research, Taipei Veterans General Hospital, Taipei, Taiwan, ROC.; 5 Department of Physical Medicine and Rehabilitation, Cheng Hsin General Hospital, Taipei, Taiwan, ROC.; 6 Department of Physiology and Pharmacology, College of Medicine, Chang Gung University, Taoyuan, Taiwan, ROC.; 7 Medical Center of Aging Research, China Medical University Hospital, Taichung, Taiwan, ROC.

**Keywords:** α-tomatine, cytokines, MAPK, NF-κB, splenocytes

## Abstract

α-Tomatine is a steroidal glycoalkaloid found in immature tomatoes that has been shown to have multiple beneficial effects on health. However, its anti-inflammatory properties have been little investigated thus far. The aim of this study was to evaluate its potential anti-inflammatory properties and underlying molecular mechanisms in rat splenocytes *in vitro* and *ex vivo*. We measured the lipopolysaccharide (LPS)-stimulated secretion of inflammatory molecules by splenocytes to assess the anti-inflammatory effects of α-tomatine. The underlying mechanism was investigated *via* western blotting. Next, we verified the anti-inflammatory potential of α-tomatine in rat splenocytes *ex vivo*. Rats were subcutaneously injected with one of two dosages of α-tomatine for seven days. We then collected their splenocytes and used them to further investigate anti-inflammatory responses *ex vivo*. α-Tomatine reduced LPS-evoked TNF-α, IL-1β, and NO secretion in a dose-dependent manner in the splenocytes. It also suppressed the expression of phosphorylated p38, ERK, and NF-κB *in vitro*. Notably, in the *ex vivo* experimental model, α-tomatine strongly inhibited parts of the MAPK and NF-κB signaling pathways, resulting in reduced secretion of inflammatory molecules. These results revealed that α-tomatine exerted strong anti-inflammatory activities both *in vitro* and *ex vivo*. Further, its underlying mechanisms may be related to suppressing parts of the MAPK and NF-κB signaling pathways. We thus expect α-tomatine to be developed as a novel therapeutic candidate for the treatment of inflammation-related diseases.

## Introduction

The inflammatory response triggered by pathogenic infections or tissue damage is a highly complex process encompassing molecular, cellular, and physiological transformations. Inflammation is a vital physiological mechanism, serving as the body's defense against microbial infections and method of removal of harmful stimuli and restoration of cells to their normal state 1. However, excessive inflammation or inflammation in response to nonpathogenic stimuli can have serious detrimental effects on health. Central to this inflammatory process are immune cells such as macrophages, dendritic cells, and lymphocytes. These cells play a critical role by activating signaling pathways associated with inflammation, including those of toll-like receptor 4 (TLR4), mitogen-activated protein kinase (MAPK), and nuclear factor kappa B (NF-κB). These pathways orchestrate the release of large quantities of cytokines, including tumor necrosis factor α (TNF-α) and the interleukin family 2-7. These cytokines represent significant targets in the development of anti-inflammatory medications 8. In other words, inhibiting excessive release of inflammatory substances is emerging as an effective strategy for mitigating inflammation, prompting ongoing efforts by scientists to devise targeted anti-inflammatory drugs.

The accelerating pace of modern life and a deteriorating environment make people increasingly vulnerable to stress, sleep deprivation, poor diet, and pathogens, all of which can induce inflammation 9. Currently, nonsteroidal anti-inflammatory drugs (NSAIDs) are the primary clinical treatment. However, prolonged NSAID use carries the risk of severe side effects, including gastrointestinal, renal, and hepatic damage 10. Consequently, scientists are actively developing new, more effective anti-inflammatory medications and foods. Recent research has focused on natural products that are anti-inflammatory, cost-effective, and associated with fewer side effects 9. Despite the abundance of plant resources, only a small fraction have been thoroughly explored. Therefore, elucidating the mechanisms underlying their effects is crucial to understanding their potential health benefits.

Research has shown that alkaloids, which are common in plants, possess anti-inflammatory benefits, making them highly promising potential candidates for the development of new anti-inflammatory drugs or functional health foods 11, 12. This inherent anti-inflammatory activity highlights a critical area of pharmacological research aimed at leveraging natural compounds for therapeutic purposes. One specific steroidal alkaloid, α-tomatine, is primarily isolated from unripe green tomatoes 13. While recent studies have primarily emphasized α-tomatine's anticancer effects 14, which include reducing angiogenesis, cell invasion 15, 16, and cell proliferation and promoting apoptosis 16-18, there has been relatively limited research on its anti-inflammatory properties. Previous evidence has suggested that it can reduce the secretion of cytokines by inhibiting the extracellular signal-regulated kinase (ERK) and NF-κB pathways in macrophages 19. However, this initial evidence suffers from two key limitations: it stems solely from cellular studies (in vitro) and requires pharmacological doses (micromolar) to be effective. As a result, the scientific support for the anti-inflammatory effects of α-tomatine remains relatively weak and requires validation. Consequently, the foundational questions persist regarding α-tomatine's actual contribution to anti-inflammatory effects *in vivo* mammalian systems. Recently, researchers have strongly emphasized the need for further, comprehensive research into the cellular and molecular mechanisms by which α-tomatine works to gain a more complete understanding of its true effects and therapeutic potential on human health 11. A deeper mechanistic understanding is essential to bridge the gap between preliminary cellular findings and practical clinical applications.

In this study, therefore, we used rat splenocytes as a research model for a more in-depth investigation of the anti-inflammatory effects and mechanisms of α-tomatine in *in vitro* and *ex vivo* experiments. Based on previous research, we hypothesized that α-tomatine would attenuate the lipopolysaccharide (LPS)-evoked inflammatory responses via MAPK/NF-κB signaling in these experiments.

## Materials and Methods

### Animals

The male Sprague-Dawley rats used in all experiments were obtained from the Laboratory Animal Center of National Yang Ming Chiao Tung University were housed in plastic cages, maintained in an air-conditioned atmosphere under a 14 h light/dark (05:00-19:00) cycle, and provided with free access to tap water and standard chow. The rats were randomly divided into three groups (n=5 each). The control group received subcutaneous injections of sesame oil (1 ml/kg body weight; Sigma-Aldrich, St. Louis, MO, USA) once daily for seven consecutive days. In contrast, the experimental groups were administered subcutaneous injections of α-tomatine (0.4 or 2 mg/kg body weight; Santa Cruz Biotechnology, Dallas, TX, USA) once daily for seven days prior to sacrifice by decapitation. The rats were then sacrificed and the spleen was rapidly excised, and the splenocytes were prepared for *in vitro* and *ex vivo* experiments. All animal studies were approved by the Institutional Animal Care and Use Committee of National Yang Ming Chiao Tung University under approval number 1050611.

### Preparation and culture of splenocytes

The splenocytes were prepared according to methods described previously 20. The spleens were excised from the rats and homogenized in RPMI-1640 medium supplemented with 2 mM l-glutamate, 25 mM HEPES, 100 U/ml penicillin, 100 μg/ml streptomycin, 50 μM β-mercaptoethanol, and 10% heat-inactivated (56 °C, 30 min) fetal bovine serum (FBS, Sigma-Aldrich, St. Louis, MO, USA). Cell mixtures were filtered through a 0.70 μm sterile nylon mesh screen and the cells were collected *via* centrifugation. The red blood cells were lysed by adding ammonium-chloride-potassium (ACK) lysing buffer (0.15 M NH_4_Cl, 1 mM KHCO_3_, and 0.1 mM Na_2_-EDTA; pH 7.4). Next, after they had been washed twice with phosphate-buffered saline (PBS), the splenocytes were finally resuspended in medium and the number of live cells was calculated by staining them with trypan blue. The primary splenocytes were then adjusted to a concentration of 4 × 10^6^ cells/ml and loaded into culture plates. For the cell culturing experiments, the cells were stimulated LPS in the presence or absence of α-tomatine at 37 °C in a humid atmosphere of 5% CO_2_. The supernatants and cells were collected for investigation of their inflammatory response and the molecular mechanisms involved.

### Cytokine assays

The levels of pro-inflammatory cytokines in the supernatants of the cultured splenocytes were assessed using commercial enzyme-linked immunosorbent assay (ELISA) kits specific to rat cytokines (eBioscience, San Diego, CA, USA) according to the manufacturer's instructions. The optical density (O.D.) was detected using a microplate reader and normalized to two million cells/well in both the *in vitro* and *ex vivo* experiments.

### MTT assays

An MTT (3-[4, 5-dimethylthiazol-2-yl]-2, 5-diphenyltetrazolium bromide) (Sigma-Aldrich) assay was performed to assess the cytotoxicity of α-tomatine and LPS-stimulated cell proliferation toward the splenocytes. Splenocytes (2 × 10^5^/well) were loaded into 96-well flat-bottom plates with different concentrations of α-tomatine (10^-11^ to 10^-7^ M) with or without LPS (Sigma-Aldrich) and incubated for 24 h at 37 °C under 5% CO_2_. An MTT solution (20 μl) was added to each well, followed by incubation for 4 h. At the end of this incubation period, 100 μl of stop solution (10% SDS-0.01 N HCl) was added to each well, and the O.D. was read at 570 nm and 630 nm using an immunosorbent assay plate reader.

### Nitrite determination

The splenocytes were cultured in 96-well plates at 4 × 10^5^ cells/well and incubated with culture medium (vehicle) or LPS (10 μg/ml) in the presence or absence of tomatine (10^-10^ to 10^-7^ M) at 37 °C in a humid atmosphere of 5% CO_2_ for 24 h. The NO level was determined via Griess reagent (Sigma-Aldrich) quantification of nitrites (relatively stable metabolites of NO) according to the manufacturer's recommendations. In brief, 100 μl of splenocyte culture medium was mixed with an equal volume of Griess reagent in a 96-well plate and incubated for 15 min at room temperature, then subjected to spectrophotometric analysis at a wavelength of 540 nm using a microplate reader. The nitrite concentrations were calculated by comparison with an NaNO_2_ standard curve for the range 0-100 μM.

### Western blot analysis

The sample of splenocytes was homogenized in triple detergent lysis buffer (50 mM Tris-HCl [pH 8.0], 150 mM NaCl, 0.02% sodium azide, 0.5% sodium deoxycholate, 1% Nonidet P-40, 0.1% SDS, 100 μg/ml PMSF, 1 μg/ml aprotinin, and complete protease inhibitor cocktail). The protein concentrations of the homogenates were measured *via* Bradford protein assay. The extracted proteins (50 μg) were electrophoretically fractionated using SDS-polyacrylamide gels and then electrotransferred to polyvinylidene fluoride membranes (Millipore, Billerica, MA, USA). The membranes were incubated for 1 h with blocking buffer (Tris-buffered saline [TBS]-T buffer containing 3% bovine serum albumin) at room temperature. Next, the membranes were incubated overnight at 4 °C with the following specific primary antibodies: TLR4 (Imgenex, San Diego, CA, USA), MAPK (Cell Signaling Technology, Danvers, MA, USA) and NF-κB (Cell Signaling Technology). After extensive washing with TBS-T buffer, horseradish peroxidase-conjugated secondary antibodies were applied to the blots and incubated at room temperature for 1 h. The specific signals were detected using an enhanced chemiluminescence reagent (PerkonElmer Life Science, Boston, MA, USA) and imaged using the LAS 4000 imaging system (Fuji Film, Tokyo, Japan).

### Statistical analysis

Data are presented as mean ± standard error of the mean (SEM). The results were analyzed *via* one-way analysis of variance (ANOVA) using SPSS 20.0 software, followed by Bonferroni *post hoc* tests 21. A *P* value of < 0.05 was considered statistically significant.

## Results

### Cytotoxic effects of α-tomatine on splenocytes

**Figure 1:**
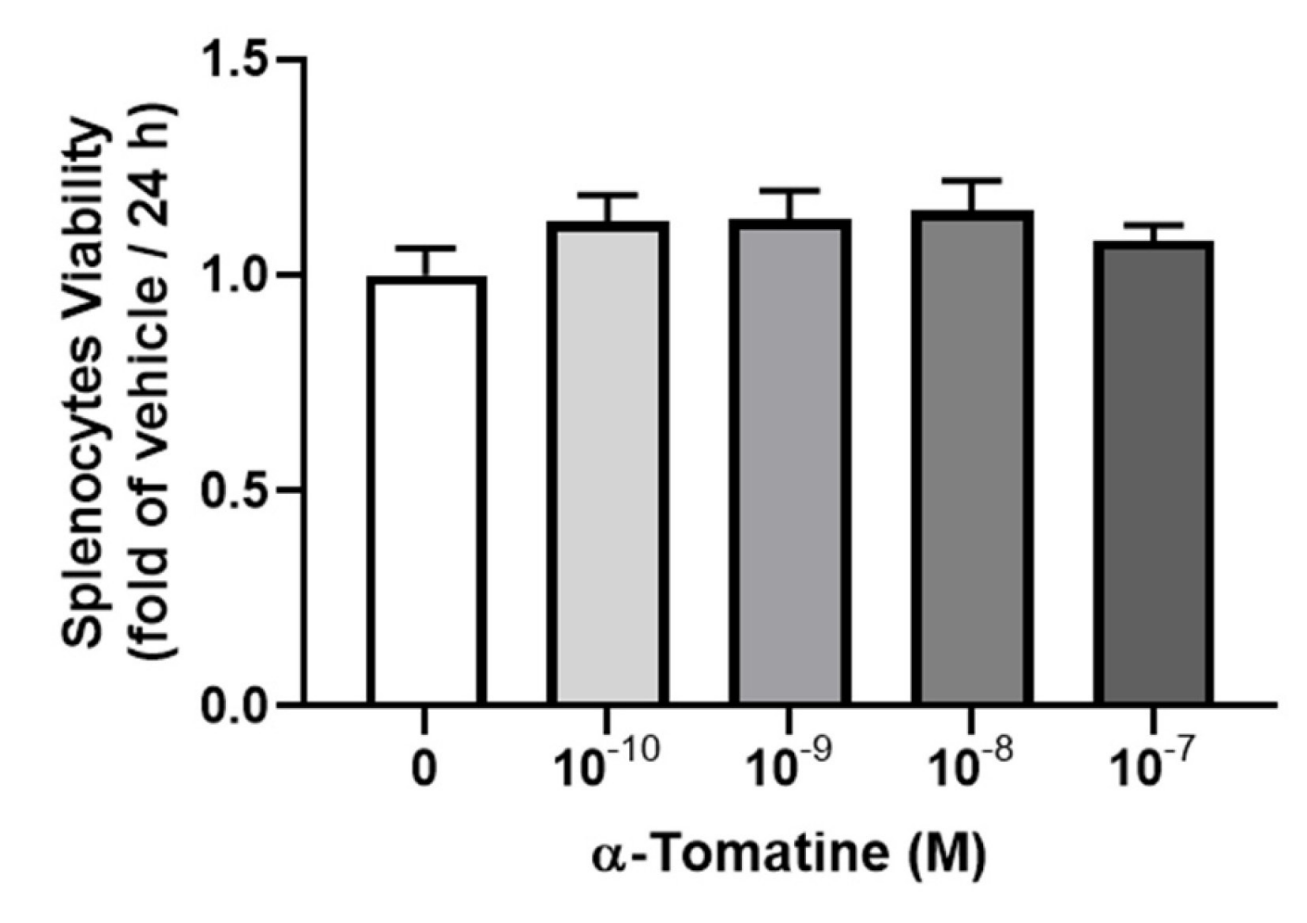
** Cytotoxic effects of α-tomatine on rat splenocytes.** The cells were treated with α-tomatine (10^-10^ to 10^-7^ M) for 24 h and cell viability was measured via the MTT assay. No significant differences in cell viability were observed at any concentration tested, indicating that α-tomatine does not exert cytotoxic effects on splenocytes within this dose range. Data are expressed as mean ± SEM (n=5).

We examined the cytotoxicity of α-tomatine toward splenocytes at various dosages, from 10^-10^ to 10^-7^ M, by visualizing the reduction in tetrazolium salts (MTT assay) over 24 h. As shown in Figure 1, α-tomatine did not significantly alter cell viability at any concentration, whether or not 10 µg/ml of LPS had been added. These results suggest that α-tomatine exhibited no toxicity toward splenocytes at concentrations of up to 10^-7^ M. All subsequent experiments were carried out using concentrations of 10^-7^ M or less.

### Effects of α-tomatine on inflammatory-molecule production in splenocytes

**Figure 2:**
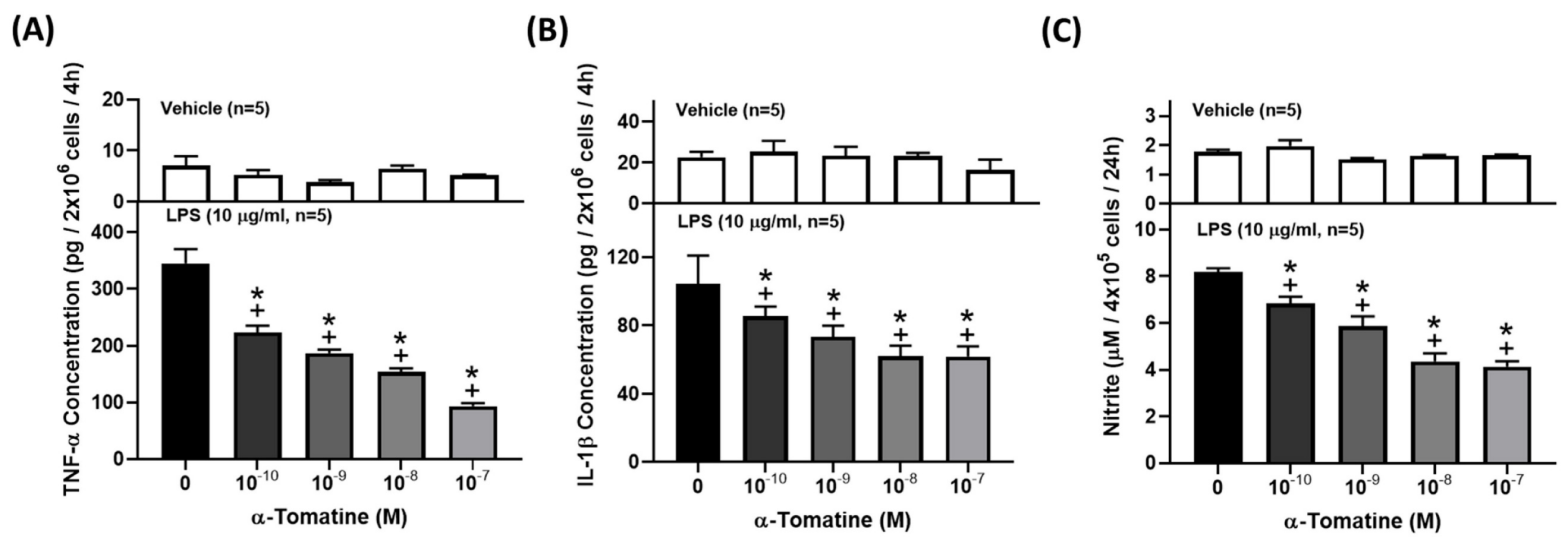
** Effects of α-tomatine on the production of TNF-α (A), IL-1β (B), and NO (C) in LPS-stimulated splenocytes.** Splenocytes were treated with α-tomatine (10^-10^ to 10^-7^ M) and with (lower panel) or without (upper panel) LPS (10 μg/ml) for 4 h. Cytokines and NO concentration were measured using ELISA kits and Griess reagent, respectively. α-Tomatine reduced LPS-evoked TNF-α, IL-1β, and NO secretion in a dose-dependent manner in the splenocytes. Data are expressed as mean ± SEM (n=5). ^+^ *P* < 0.05, compared with vehicle group. * *P* < 0.05, compared with LPS-alone group.

Cytokines and NO are well-known inflammatory mediators that play a fundamental role in the pathogenesis of inflammation-related diseases. To investigate the anti-inflammatory effects of α-tomatine on LPS-stimulated splenocytes, we determined the concentrations of TNF-α, IL-1β, and NO in the culture media using ELISA kits or Griess reagent. As shown in Figures 2A and 2B, exposure of splenocytes to LPS for 4 h dramatically induced the production of pro-inflammatory cytokines (TNF-α and IL-1β), and the production of cytokines decreased in a dose-dependent manner with the addition of α-tomatine. Similarly, LPS significantly upregulated the secretion of NO by splenocytes, and this was reduced in α-tomatine-treated cells (Fig. 2C).

### Effects of α-tomatine on expression of inflammatory signaling pathways in LPS-induced splenocytes

**Figure 3:**
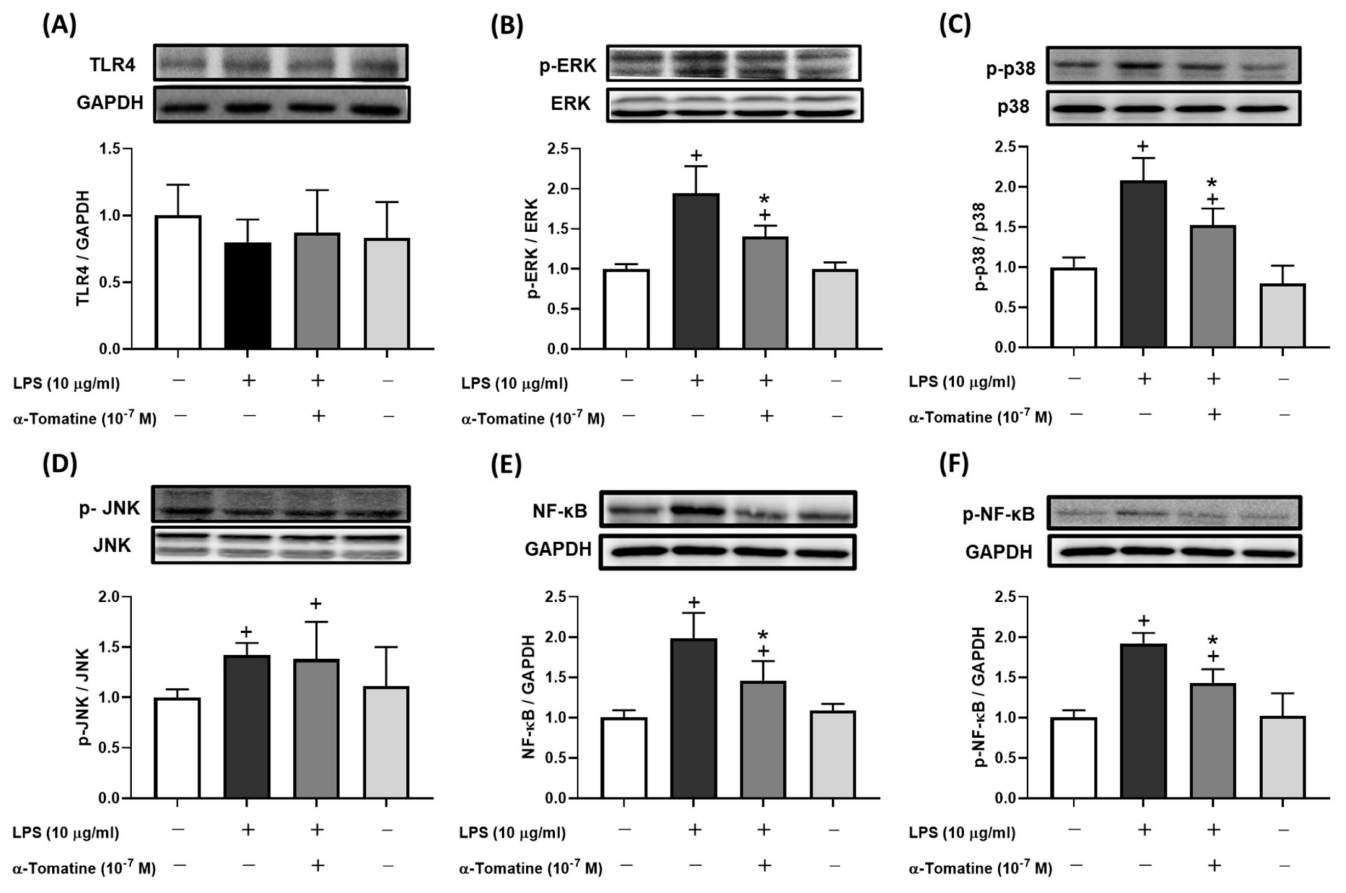
** Effects of α-tomatine on expression of LPS-induced inflammatory signaling pathway in splenocytes.** Splenocytes were treated with LPS (10 μg/ml) alone or co-treated with α-tomatine (10^-7^ M) for 4 h. Western blot analysis was performed to examine the expression levels of TLR4 (A), p-ERK (B), p-p38 (C), p-JNK (D), total NF-κB (E), and phosphorylated NF-κB (p-NF-κB, F). LPS stimulation significantly increased the phosphorylation levels of ERK, p38, JNK, and NF-κB. Co-treatment with α-tomatine effectively attenuated the LPS-induced phosphorylation of ERK, p38, and NF-κB, but did not affect the elevated p-JNK levels. The expression level of TLR4 remained unchanged across all treatment groups. Representative Western blot images are shown above the corresponding quantitative bar graphs. Data are presented as mean ± SEM (n = 5). ^+^ *P* < 0.05, compared with control group. * *P* < 0.05, compared with LPS-alone group.

To further investigate α-tomatine's inhibition of inflammatory mediators, we examined its suppression of the corresponding synthase pathways by measuring the protein expression of TLR4, MAPK, and NF-κB. The western blot analyses showed that levels of MAPK and NF-κB protein were considerably upregulated in LPS-stimulated splenocytes compared to controls (Fig. 3), whereas there was no significant difference in TLR4 expression (Fig. 3A). As shown in Figures 3B-3D, phosphorylation of ERK and p38 was attenuated in splenocytes treated with α-tomatine (10^-7^ M) and stimulated with LPS, but there was no significant difference in the phosphorylation of c-Jun N-terminal kinases (JNK). Interestingly, expression of NF-κB and its phosphorylation were similarly reduced by α-tomatine at a concentration of 10^-7^ M in LPS-stimulated splenocytes (Fig. 3E and 3F). These results suggest that the inhibitory effects of α-tomatine on these aspects of the MAPK and NF-κB signaling pathways are consistent with its inhibitory effects on inflammatory responses in LPS-stimulated splenocytes.

### Anti-inflammatory effects of α-tomatine in LPS-induced inflammatory responses in rat *ex vivo*

**Figure 4:**
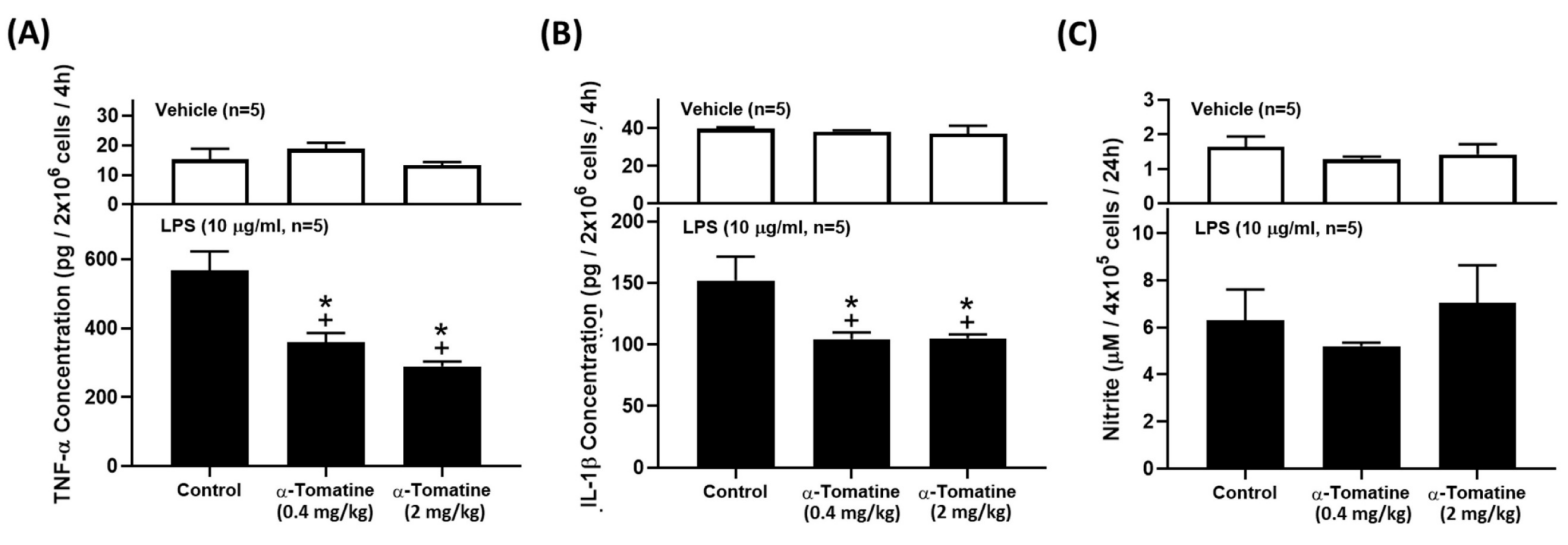
** α-Tomatine attenuated LPS-evoked inflammatory mediators in splenocytes *ex vivo.*** Rats were subcutaneously administered α-tomatine (0.4 or 2.0 mg/kg) once daily for seven days. The splenocytes were harvested and treated *ex vivo* with (lower panel) or without (upper panel) 10 μg/ml of LPS for 4 h. Cytokines and NO concentration were measured using ELISA kits and Griess reagent, respectively. LPS stimulation markedly increased cytokine and NO production. Co-treatment with α-tomatine (0.4 mg/kg or 2.0 mg/kg) significantly suppressed LPS-induced cytokine production. However, α-tomatine had no effect on LPS-induced NO production. Data are expressed as mean ± SEM (n=5). ^+^ *P* < 0.05, compared with vehicle group. * *P* < 0.05, compared with LPS-alone group.

**Figure 5:**
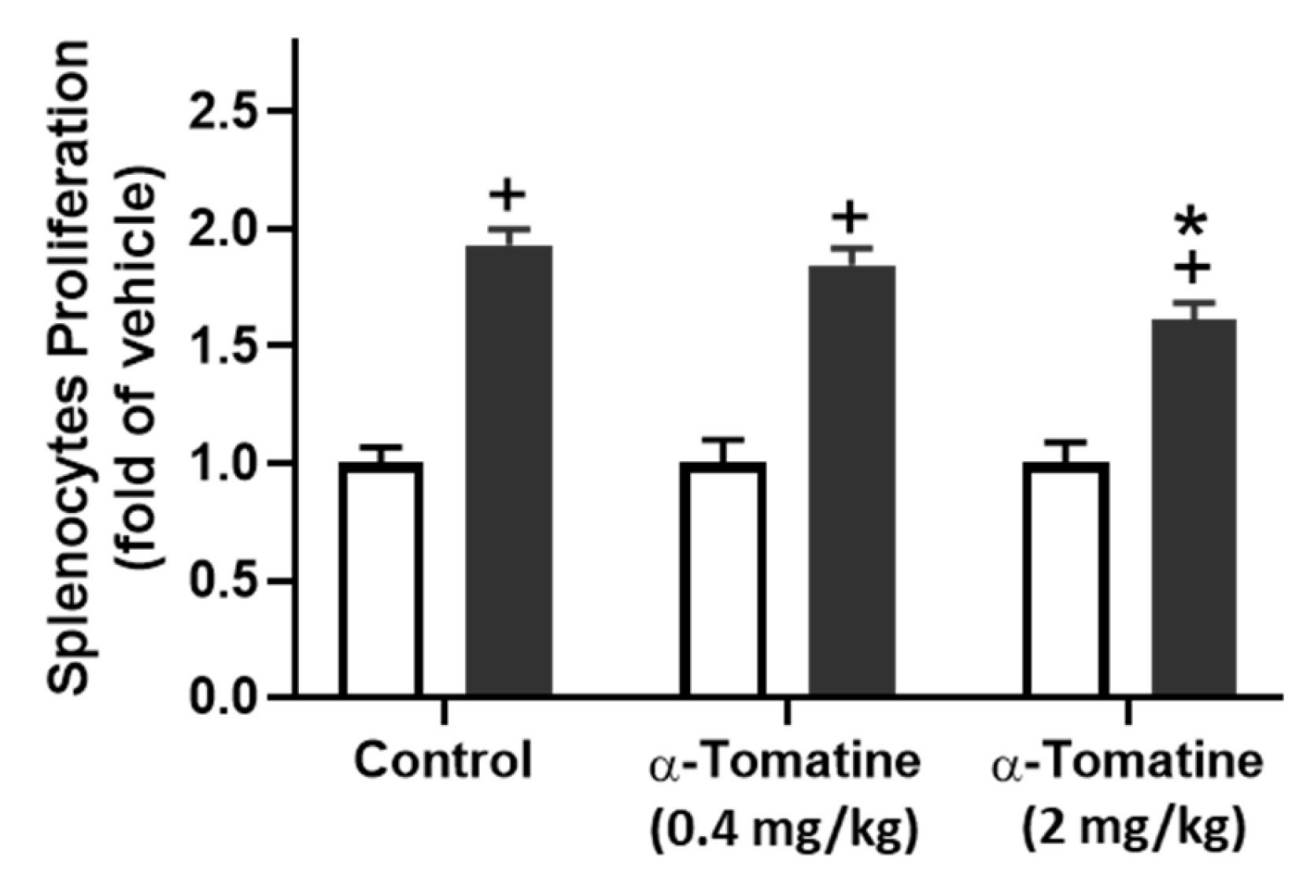
** Effect of α-tomatine on LPS-induced proliferation in splenocytes isolated from rat *ex vivo*.** Rats were subcutaneously injected with α-tomatine at concentrations of 0.4 or 2.0 mg/kg once daily for seven days. The splenocytes were harvested and exposed to 10 μg/ml of LPS for 24 h. Splenocyte proliferation was determined *via* MTT assay. LPS significantly enhanced splenocyte proliferation compared to the non-treated group. This LPS-induced proliferation was significantly suppressed by 2.0 mg/kg α-tomatine, whereas the lower dose (0.4 mg/kg) showed no inhibitory effect. Data are expressed as mean ± SEM (n=5).^ +^ *P* < 0.05, compared with non-treated group. * *P* < 0.05, compared with LPS group.

Because α-tomatine effectively inhibited the LPS-induced secretion of inflammatory molecules in splenocytes, we extended the experiment to determine whether α-tomatine exerted an anti-inflammatory effect in an animal model *ex vivo*. The rats were treated with α-tomatine (0.4 or 2.0 mg/kg body weight) *via* once-daily subcutaneous injection for seven days. The α-tomatine suppressed the LPS-evoked inflammatory response in the splenocytes *ex vivo*. As shown in Figure 4, exposure to LPS led to a clear inflammatory response in all groups. The anti-inflammatory effect of both doses of α-tomatine was detectable macroscopically as a significant reduction in TNF-α and IL-1β expression relative to the untreated group (Fig. 4A and 4B). Further, these beneficial effects were accompanied by a reduced splenocyte cell-proliferation ratio relative to untreated animals (Fig. 5). However, the α-tomatine did not affect LPS-induced NO production (Fig. 4C).

### Anti-inflammatory effects of α-tomatine on expression of inflammatory signaling pathways in rat *ex vivo*

**Figure 6:**
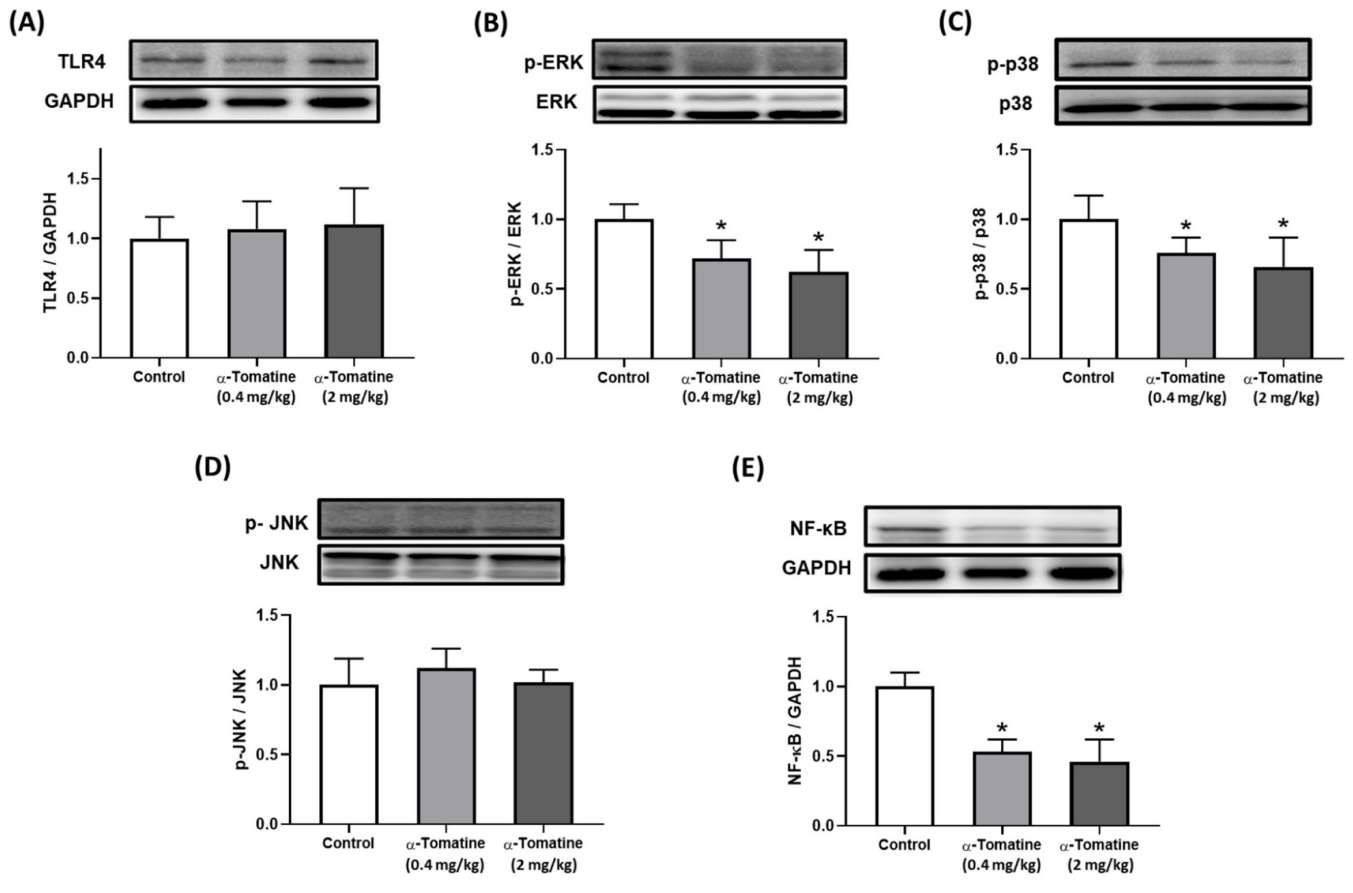
** Effects of α-tomatine on inflammatory signaling pathway expression in splenocytes *ex vivo*.** Rats were subcutaneously injected with α-tomatine at concentrations of 0.4 or 2.0 mg/kg once daily for seven days. Splenocytes were then isolated, homogenized, and subjected to Western blot analysis to evaluate the expression of TLR4 (A), p-ERK (B), p-p38 (C), p-JNK (D), and NF-κB (E). Compared to the control group, both doses of α-tomatine significantly reduced the expression levels of p-ERK, p-p38, and NF-κB, indicating inhibition of inflammatory signaling. However, no significant changes were observed in TLR4 and p-JNK expression. There were no significant differences between the two α-tomatine doses. Representative blot images are shown above the corresponding quantitative bar graphs. Data are expressed as mean ± SEM (n=5). * *P* < 0.05, compared with control group.

Our biochemical analysis of the splenocytes confirmed the anti-inflammatory effects of α-tomatine in our *ex vivo* experimental model. According to our *in vitro* data, the anti-inflammatory effects of α-tomatine may be associated with the blockade of the TLR4 downstream signaling pathway. We therefore investigated the involvement of the TLR4 signaling-related pathway in the splenocytes of α-tomatine-treated rats *via* western blot. As shown in Figure 6, there were significant differences between the vehicle- and α-tomatine-treated groups in terms of the MAPK pathway. First, regarding TLR4 protein expression, α-tomatine had effects similar to those seen *in vitro*, showing no significant change in TLR4 expression (Fig. 6A). Treatment with α-tomatine (0.4 and 2.0 mg/kg) strongly reduced the phosphorylation of ERK and p38 relative to the vehicle group (Fig. 6B and 6C). However, α-tomatine did not significantly affect the phosphorylation level of JNK (Fig. 6D). Additionally, pretreatment with α-tomatine decreased the expression level of NF-κB compared to the control group (Fig. 6E).

## Discussion

Previous research, using macrophage cell line RAW264.7, indicate that α-tomatine has anti-inflammatory effects *via* the inhibition of ERK and NF-κB expression 19. However, its effects on several other signaling pathways involved in the intracellular inflammatory response are as yet unknown. It is important to investigate this, considering that these pathways may be potential therapeutic targets in the treatment of inflammatory disease. In the present study, we examined the anti-inflammatory activity and other putative inhibitory mechanisms of α-tomatine in primary splenocytes *in vitro* and *ex vivo*. First, we assessed its effects at concentrations of ≤ 10^-7^ M on splenocyte viability and found that it exhibited no cytotoxicity. Next, we assessed its effects on various inflammation-related proteins and pathways. While it had no effect on TLR4 expression, it did have an inhibitory effect on the expression of phosphorylated ERK, p38, and NF-κB, indicating that its inhibitory effect on cytokine production is linked to partial inhibition of the MAPK signaling pathway. We then confirmed the inhibitory activity of α-tomatine administration against the secretion of LPS-induced inflammatory molecules (TNF-α, IL-1β, and NO) in splenocytes. This is the most basic parameter for evaluating anti-inflammatory activity *ex vivo*. We also then explored the TLR4 downstream signaling pathway. Our data show that α-tomatine treatment significantly reduced the LPS-evoked secretion of TNF-α and IL-1β *via* the expression of phospho-ERK and p38 *ex vivo*. Combined, these findings show that α-tomatine can function as a potent inflammation inhibitor.

Splenocytes include T and B cells, monocytes, dendritic cells, and other types of immune cells, all of which are crucial for mediating the inflammatory response 22. Lipopolysaccharide is an endotoxin capable of triggering an intense robust immune response in splenocytes, and it is commonly used in research to stimulate inflammatory reactions and facilitate the development of anti-inflammatory foods and drugs 23-25. It triggers splenocytes to activate TLR4 and downstream inflammation-related signaling pathways, including MAPK and NF-κB. This activation leads to the synthesis of various inflammatory molecules, including TNF-α, IL-1β, and NO, which are the basis of many inflammatory-related diseases. However, over the past decade, only one study has reported the effective reduction of LPS-induced cytokine secretion in macrophages by α-tomatine 19. This was achieved through the inhibition of iNOS expression, ERK phosphorylation, and translocation of NF-κB. Despite this, research evidence supporting the further development of α-tomatine as an anti-inflammatory health food or drug remains limited. In addition, that study required a high concentration (10^-6^ M) of α-tomatine to achieve the observed effects 19. In the present study, we used splenocytes as the research model, and we found that while α-tomatine did not alter TLR4 expression, it did reduce the secretion of inflammatory molecules by modulating the TLR4 downstream signaling pathways, including MAPK and NF-κB. These findings align with prior research findings, suggesting that α-tomatine holds promise as a candidate for the development of anti-inflammatory foods and drugs.

When immune cells are exposed to LPS, it activates the MAPK and NF-κB signaling pathways, which are known to be pivotal in regulation of the immune response and inflammation 2, 3, 20, 23, 26. The three primary members of the MAPK family—ERK, JNK, and p38—are recognized as important intracellular responders that are part of a signaling cascade that plays a role in inflammatory illnesses 27. Activation of MAPK results in the phosphorylation of all three of these proteins, which promotes the inflammatory response by facilitating the synthesis and release of numerous inflammatory mediators. As mentioned above, a study involving macrophages has demonstrated that at a concentration of 10^-6^ M, α-tomatine reduce TNF-α synthesis by inhibiting ERK phosphorylation 19. Our findings indicated that even lower concentrations of α-tomatine can effectively exert an anti-inflammatory effect. Apart from modulating ERK, α-tomatine also exhibits the ability to regulate cytokine secretion in splenocytes, by inhibiting p38 phosphorylation.

Moreover, when the cells are at rest, NF-κB attaches to the IκB protein in the cytoplasm and exists in an inactive form 28. However, following stimulation by LPS, IκB is activated and degrades, and the NF-κB then translocates into the nucleus and increases the activation of other pro-inflammatory genes 29, 30. MAPK serves as the upstream signaling pathway of NF-κB, with previous research indicating that inhibition of phospho-p38 leads to attenuation of the nuclear translocation of NF-κB p65 31, 32.

We hypothesize that α-tomatine may disrupt the inflammatory response *via* the MAPK/NF-κB signaling pathway. To investigate this hypothesis, we conducted western blot experiments, which revealed that α-tomatine significantly reduces the phosphorylation of p65 in splenocytes. This confirms that the reduction of p38 expression can lead to the downregulation of NF-κB signaling, thereby eliciting anti-inflammatory effects. In contrast to the study that used macrophage cell lines 19, whereas the present study used splenocytes. Splenocytes include a diverse array of immune cells, allowing for a more comprehensive understanding of the role of α-tomatine in anti-inflammatory responses. The findings of this study hold substantial practical implications for the future, extending the findings of previous research while providing additional scientific evidence to affirm the beneficial effects of α-tomatine.

Recent research has demonstrated the potent anti-cancer effects of α-tomatine with minimal associated side effects 18. Prior toxicology studies have also reported that oral administration of moderate quantities of α-tomatine for a duration of four weeks does not elicit any discernible toxicity in organisms 33. These findings align with the results of our cytotoxicity tests, which indicate that concentrations of up to 10^-7^ M do not exhibit cytotoxicity toward splenocytes. Consequently, α-tomatine can be regarded as a safe candidate for anti-inflammatory drug development and as a component of health-food products.

In the inflammation process, it is well known that immune cells release substantial quantities of inflammatory factors, including TNF-α, IL-6, IL-1β, and NO 2-4, 23, 24. Excessive production of these inflammatory factors result in tissue or organ damage. Therefore, an effective strategy for mitigating the inflammatory response involves reducing the excessive release of inflammatory factors by immune cells. However, previous investigations about the anti-inflammatory effects of α-tomatine have been predominantly limited to *in vitro* cell experiments and have not progressed to *in vivo/ex vivo* experiments involving mammals. Consequently, the precise anti-inflammatory impact of α-tomatine *ex vivo* remains to be clarified. In our study, we administered subcutaneous injections of α-tomatine at doses of 0.4 and 2.0 mg/kg to rats for seven consecutive days. Subsequently, we induced an acute inflammatory response in their splenocytes *ex vivo* through LPS administration. The results revealed that rats pre-treated with α-tomatine exhibited significant inhibition of their secretion of TNF-α and IL-1β, with the signaling regulation pathway mirroring the *in vitro* experiment. Interestingly, α-tomatine reduced LPS-induced NO production *in vitro* but not *ex vivo*, suggesting potential differences in iNOS regulation between isolated splenocytes and those derived from α-tomatine-treated rats. This discrepancy may be attributable to systemic physiological factors present ex* vivo*, such as circulating metabolites of α-tomatine or interactions with other immune cells, which could modulate iNOS activity. To elucidate the physiological basis of this difference, future studies should assess LPS-induced iNOS expression and activity in splenocytes *ex vivo*. Moreover, although no significant difference was observed between the doses, these outcomes provide substantial evidence supporting the robust anti-inflammatory potential of α-tomatine as a promising candidate for anti-inflammatory drug development.

Notwithstanding the valuable findings of this study, certain limitations should be acknowledged. First, the use of splenocytes offers a comprehensive model of immune interactions but their cellular heterogeneity complicates identifying specific mechanisms, and the findings' relevance to human inflammation requires validation with human cells or clinical samples. Future studies should complement splenocyte data with purified cell or in vivo models for clearer mechanistic and translational insights. Second, while α-tomatine is biologically active, it is associated with some toxicity whose mechanisms are not fully elucidated. Additional mechanistic studies are necessary to characterize its toxicological profile and develop strategies to attenuate adverse effects, thereby optimizing its therapeutic applicability 34. Third, present study lacked a positive control group (e.g., dexamethasone). Although α-tomatine's anti-inflammatory effects were demonstrated, the absence of a direct comparison with a known agent makes it challenging to quantitatively evaluate its relative efficacy and therapeutic potency. Future research should incorporate a positive control to establish a robust benchmark for assessing its clinical relevance.

**Figure 7:**
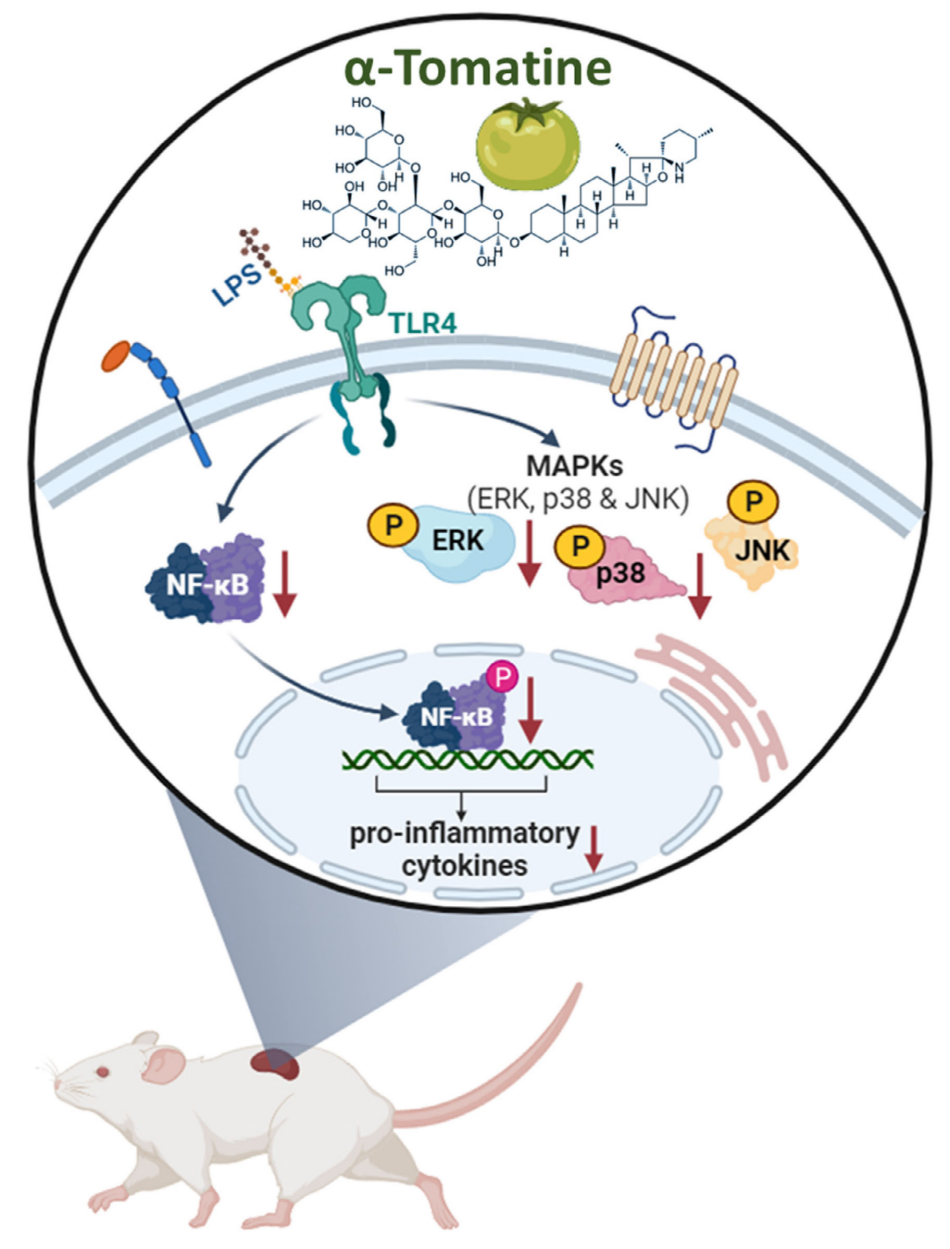
** Schematic summary of the anti-inflammatory activities exerted by α-tomatine in splenocytes.** Our findings suggest that α-tomatine significantly abrogates the LPS-evoked secretion of inflammatory molecules and that the underlying mechanisms may be related to suppressing parts of the MAPK and NF-κB signaling pathways. These results suggest that α-tomatine may have potent benefits for modulating the immune system.

This research shows that α-tomatine exerted anti-inflammatory activities in LPS-stimulated splenocytes *in vitro* and *ex vivo*. The action mechanism behind α-tomatine effects was that it suppressed the LPS-evoked secretion of inflammatory molecules by attenuating the levels of the MAPK (ERK and p38)/NF-κB signaling pathways (Fig. 7). As an adjuvant medication or dietary additive, α-tomatine may be useful for preventing or treating inflammation.
